# Delayed Diagnosis and Complications of Predominantly Antibody Deficiencies in a Cohort of Australian Adults

**DOI:** 10.3389/fimmu.2018.00694

**Published:** 2018-05-14

**Authors:** Charlotte A. Slade, Julian J. Bosco, Tran Binh Giang, Elizabeth Kruse, Robert G. Stirling, Paul U. Cameron, Fiona Hore-Lacy, Michael F. Sutherland, Sara L. Barnes, Stephen Holdsworth, Samar Ojaimi, Gary A. Unglik, Joseph De Luca, Mittal Patel, Jeremy McComish, Kymble Spriggs, Yang Tran, Priscilla Auyeung, Katherine Nicholls, Robyn E. O’Hehir, Philip D. Hodgkin, Jo A. Douglass, Vanessa L. Bryant, Menno C. van Zelm

**Affiliations:** ^1^Department of Clinical Immunology and Allergy, The Royal Melbourne Hospital, Melbourne, VIC, Australia; ^2^Immunology Division, The Walter and Eliza Hall Institute for Medical Research, Melbourne, VIC, Australia; ^3^Department of Medical Biology, The University of Melbourne, Melbourne, VIC, Australia; ^4^The Jeffrey Modell Diagnostic and Research Centre for Primary Immunodeficiencies, Melbourne, VIC, Australia; ^5^Department of Allergy, Immunology and Respiratory Medicine, The Alfred Hospital, Melbourne, VIC, Australia; ^6^Department of Infectious Diseases, Monash University and Alfred Hospital, Melbourne, VIC, Australia; ^7^Department of Respiratory and Sleep Medicine, The Austin Hospital, Melbourne, VIC, Australia; ^8^Department of Medicine, Monash Medical Centre, Melbourne, VIC, Australia; ^9^Department of Allergy and Immunology, Monash Medical Centre, Melbourne, VIC, Australia; ^10^School of Medicine, The University of Melbourne, Melbourne, VIC, Australia; ^11^Department of Immunology and Pathology, Central Clinical School, Monash University and Alfred Hospital, Melbourne, VIC, Australia

**Keywords:** predominantly antibody deficiency, primary immunodeficiency, diagnostic delay, common variable immunodeficiency, X-linked agammaglobulinemia, immunoglobulin subclass deficiency, specific antibody deficiency

## Abstract

**Background:**

Predominantly antibody deficiencies (PADs) are the most common type of primary immunodeficiency in adults. PADs frequently pass undetected leading to delayed diagnosis, delayed treatment, and the potential for end-organ damage including bronchiectasis. In addition, PADs are frequently accompanied by comorbid autoimmune disease, and an increased risk of malignancy.

**Objectives:**

To characterize the diagnostic and clinical features of adult PAD patients in Victoria, Australia.

**Methods:**

We identified adult patients receiving, or having previously received immunoglobulin replacement therapy for a PAD at four hospitals in metropolitan Melbourne, and retrospectively characterized their clinical and diagnostic features.

**Results:**

179 patients from The Royal Melbourne, Alfred and Austin Hospitals, and Monash Medical Centre were included in the study with a median age of 49.7 years (range: 16–87 years), of whom 98 (54.7%) were female. The majority of patients (116; 64.8%) met diagnostic criteria for common variable immunodeficiency (CVID), and 21 (11.7%) were diagnosed with X-linked agammaglobulinemia (XLA). Unclassified hypogammaglobulinemia (HGG) was described in 22 patients (12.3%), IgG subclass deficiency (IGSCD) in 12 (6.7%), and specific antibody deficiency (SpAD) in 4 individuals (2.2%). The remaining four patients had a diagnosis of Good syndrome (thymoma with immunodeficiency). There was no significant difference between the age at diagnosis of the disorders, with the exception of XLA, with a median age at diagnosis of less than 1 year. The median age of reported symptom onset was 20 years for those with a diagnosis of CVID, with a median age at diagnosis of 35 years. CVID patients experienced significantly more non-infectious complications, such as autoimmune cytopenias and lymphoproliferative disease, than the other antibody deficiency disorders. The presence of non-infectious complications was associated with significantly reduced survival in the cohort.

**Conclusion:**

Our data are largely consistent with the experience of other centers internationally, with clear areas for improvement, including reducing diagnostic delay for patients with PADs. It is likely that these challenges will be in part overcome by continued advances in implementation of genomic sequencing for diagnosis of PADs, and with that opportunities for targeted treatment of non-infectious complications.

## Introduction

Primary immunodeficiencies (PIDs) are a heterogeneous group of diseases, characterized by an impaired immune response to pathogens, predisposing to more frequent and severe infection, in some instances to a single pathogen, and dysregulated immune function, which may result in autoimmune disease or inflammatory conditions (1). Knowledge of the key molecular processes underpinning these varied disorders continues to evolve with advances in genomic technology. The archetypal classification of immunologic disorders has recognized predominantly antibody deficiency (PAD) as the most prevalent PID, and these patients require lifelong antibody replacement therapy (2, 3). In comparison with other countries, the prevalence of PADs in Australia has not been clearly established. There are only two reports over the last two decades with varied results that are likely to represent significant under reporting, due to ascertainment bias (4, 5).

Diagnosis is challenging because PADs have varied clinical presentations and may present from infancy to late adulthood. The hallmark clinical feature is a history of recurrent sino-pulmonary bacterial infections, resulting from an ineffective antibody response, however, in many cases, non-infectious complications, such as autoimmune disease, or malignancy may complicate the clinical presentation.

The most profound PAD is agammaglobulinemia with a complete or near-complete absence of serum Ig and absence of mature B cells in blood. Most of these individuals suffer from X-linked agammaglobulinemia (XLA) which occurs due to mutations in the gene encoding Bruton’s tyrosine kinase (*BTK*), an enzyme essential for B-cell development (6). Defects in other genes crucial for B-cell development and survival have been identified in a smaller proportion of patients (7–10), currently leaving only 5–10% without a genetic diagnosis.

Common variable immunodeficiency (CVID) constitutes the majority of PAD cases which require ongoing treatment, with a global incidence of approximately 1:25,000 (11) although this varies according to the population studied. A recent Finnish study found the prevalence to be as high as 6.9 per 100,000 (12). CVID has complex clinical features and pathophysiology, underpinned by identifiable monogenic defects in a minority of patients (13–15). Patients may present with a spectrum of manifestations, including recurrent infections and autoimmune cytopenias. Several diagnostic criteria, based upon a combination of clinical and laboratory features, may be used to aid in diagnosis and classification of patients with these complex presentations (16–18). A diagnosis may be made from 2 years of age (19).

The spectrum of PAD also includes milder syndromes, albeit with variable clinical features that include selective IgA deficiency; unclassified hypogammaglobulinemia (HGG), characterized by reduced levels of IgG in the presence of normal IgA and IgM; IgG subclass deficiency (IGSCD), characterized by a reduction in one or more IgG subclasses with normal total IgG; and specific antibody deficiency (SpAD), characterized by normal serum immunoglobulins with an apparent failure to produce antibody in response to vaccines. Thymoma with immunodeficiency, otherwise eponymously known as Good syndrome, is a rare PID associated with the presence of a thymoma, although the mechanism is not well understood (1, 20–22).

We sought to identify the diagnostic approaches that had been used for PADs and potential obstacles to diagnosis. In addition we aimed to characterize the infectious and non-infectious manifestations in our patients, such as organ-specific or systemic autoimmune disease as well as malignancies such as lymphomas and gastrointestinal (GI) tumors.

## Materials and Methods

### Patients

We performed a comprehensive cross-sectional clinical analysis of adult patients with PADs managed under clinical immunology services in Victoria from January 2001 to February 2017. Patients who were currently receiving, or had previously received replacement immunoglobulin for PADs were identified by physicians specializing in Clinical Immunology at four major teaching hospitals in metropolitan Melbourne, Victoria: The Royal Melbourne Hospital, The Alfred Hospital, Monash Medical Centre and Austin Health. Patients with secondary hypogammaglobulinemia were excluded, as were individuals with a defined combined immunodeficiency, or a history of hematopoietic stem-cell transplantation. To ensure that the cohort included patients with clinically significant disease, and receiving regular follow-up, we did not include patients who were not deemed to require replacement immunoglobulin therapy. The study was carried out according to the principles of the Declaration of Helsinki and was approved by local human research ethics committees (Melbourne Health projects 2009.162, 2013.245; Walter and Eliza Hall Research Institute projects 10/02, 14/01; Alfred Health 109/15, 277/17). All living patients, or their next of kin, consented to the collection of their medical information. For those individuals who were deceased at the time of the data collection, ethical approval was obtained to review the medical records without the consent of the next of kin.

### Data Collection

Data were retrospectively collected from the clinical case notes using a pre-specified template to capture age of reported onset of symptoms, age at diagnosis and organ-specific manifestations that included respiratory, GI, neurological, musculoskeletal, organomegaly, and malignancy. We defined immune dysregulation on clinical notes reporting any of the following diagnoses: autoimmune disease, lymphoproliferative disease, organomegaly, granulomatous inflammation, and/or enteropathy.

### Statistical Analysis

We examined differences between groups using one-way ANOVA for comparison of continuous variables; Chi square analysis for comparison of categorical variables with a high frequency, and Fisher’s exact test for categorical variables with a frequency of less than 5; and Mantel–Cox log rank for comparing differences in survival between disease groups. As the data for age of onset, diagnosis and diagnostic delay were non-parametrically distributed, we have reported median values for these measures. A *p* value of less than 0.05 was considered statistically significant. Statistical analyses were performed using GraphPad Prism 7 software.

## Results

### Patient Demographics

179 patients with PAD were identified from the four centers. 98 patients were females, and 81 were males, with median age of 49 years (Table 1). CVID was the most frequent antibody deficiency disorder requiring clinical follow-up with 116 patients. XLA was diagnosed in 21 male patients. 22 patients had HGG, 4 had SpAD, and 12 were diagnosed with IGSCD. We also included four patients with thymoma and immunodeficiency, with a diagnosis of Good syndrome (Table 1).

**Table 1:** Diagnostic and demographic data of the Victorian adult predominantly antibody deficiency cohort.

Immunologic diagnosis	Number of patients (%)	Male:female ratio	Current age (years; range)	Median age at diagnosis (years)	Median age at symptom onset^a^ (years; range)	Median diagnostic delay^a^ (years; range)
All	179	0.82	49 (16–87)	36 (0–87)	20 (0–68)	7.5 (0–63)
Common variable immunodeficiency (CVID)	116 (64.8)	0.78	48.5 (16–80)	35 (2–80)	20 (1–65)	9 (1–63)
X-linked agammaglobulinemia (XLA)	21 (11.7)	N/A	31.5 (23–65)	0 (0–15)	0 (0–2)	1 (0–15)
Hypogammaglobulinemia (unclassified) (HGG)	22 (12.29)	0.16	54 (18–87)	43 (5–80)	19.5 (1–50)	4 (1–59)
Specific antibody deficiency (SpAD)	4 (2.23)	1.00	65.5 (43–81)	68 (33–72)	N/A	N/A
IgG subclass deficiency (IGSCD)	12 (6.7)	0.20	56 (30–81)	36 (7–74)	20 (2–68)	9 (6–16)
Good syndrome	4 (2.23)	3.0	69 (46–77)	60 (40–71)	58 (38–68)	2 (0–3)

*^a^Data for age at symptom onset and diagnostic delay available for the following numbers of patients in each group*.

*CVID, 83/116; XLA, 16/21; HGG, 7/22; SpAD, 1/4; IGSCD, 7/12; Good syndrome 2/4*.

### Diagnostic Features

#### Age at Diagnosis and Diagnostic Delay

**Figure 1:**
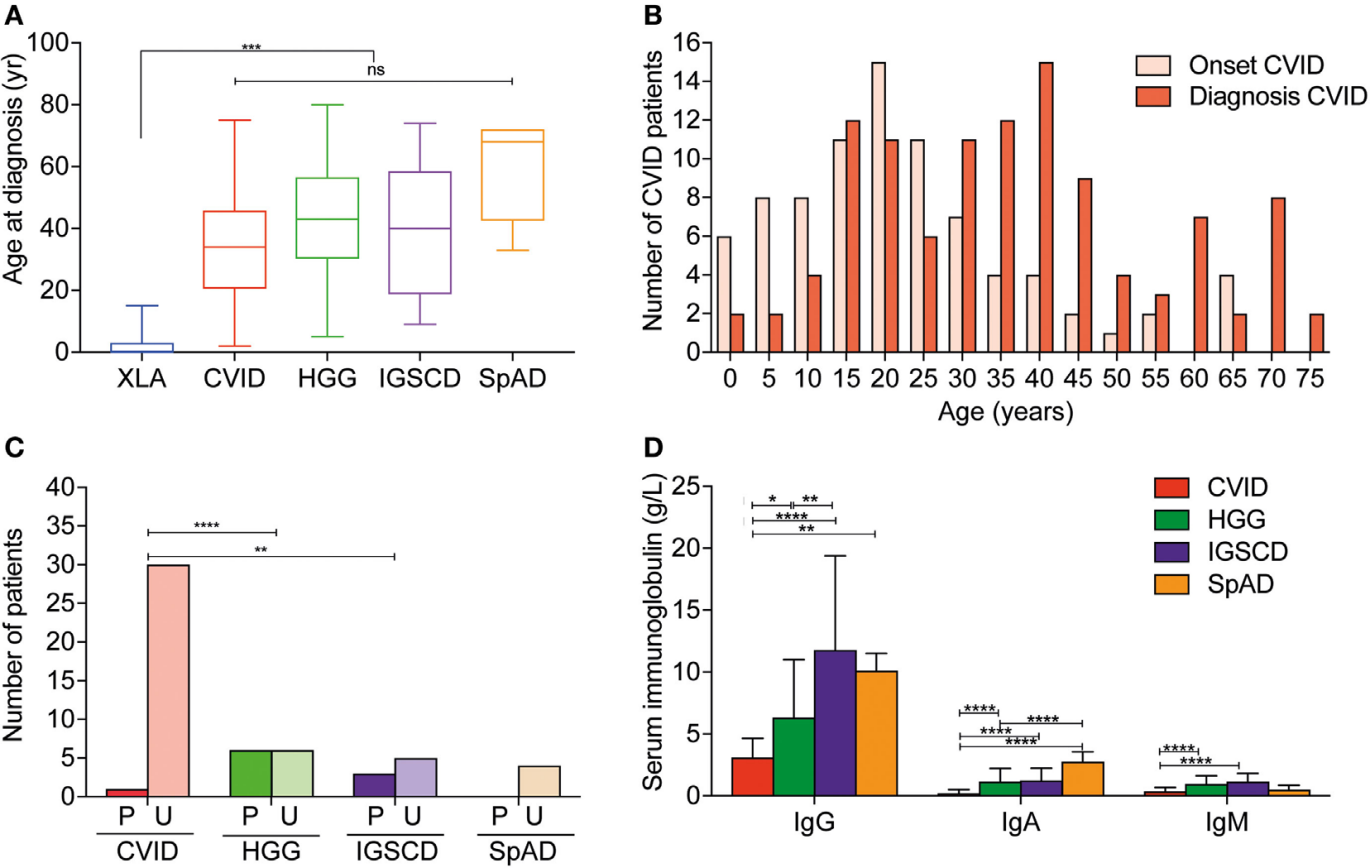
Age at diagnosis and diagnostic features differ between disease groups in Victorian adult patients with predominantly antibody deficiencies. **(A)** Box–whisker plot of age at diagnosis according to disease classification with median and 5–95% percentiles indicated. **(B)** Histogram demonstrating age at reported symptom onset, light red, and age at diagnosis (dark red) in common variable immunodeficiency (CVID) patients. **(C)** Qualitative vaccine responses to pneumococcal polysaccharide vaccine responses measured IgG binding against PPS23 polysaccharides in CVID patients (red), unclassified hypogammaglobulinemia (HGG) patients (green), IgG subclass deficiency (IGSCD) (purple), and specific antibody deficiency (SpAD) (orange). Darker colored columns indicate protective responses, P, and lighter colored columns indicate unprotective responses, U (χ^2^ analysis: **p < 0.01 and ****p < 0.0001). **(D)** Serum IgG and IgA (g/L, mean ± SD) at diagnosis according to disease classification (**p < 0.01, ***p < 0.001, and ****p < 0.0001, using one-way ANOVA).

The median age at diagnosis for the entire cohort was 36 years, which ranged from diagnoses at birth to 80 years old. Patients with XLA were diagnosed at significantly younger ages than all other antibody deficiency disorders (Figure 1A; Table 1) and experienced significantly shorter delay from symptom onset to diagnosis (Table 1; Table S1 in Supplementary Material). With regards to CVID specifically, the median age of reported onset of symptoms was 20 years, and the median age of diagnosis was 35 years (Table 1; Figures 1A,B). The median diagnostic delay was available for only 83/116 CVID patients and was 9 years. The four patients with Good syndrome were diagnosed at a median age of 60.75 years (interquartile range 44.3–74), which was significantly older than XLA (*p* < 0.0001) and CVID (*p* < 0.05) diagnoses, but not the other antibody deficiency syndromes (Table 1; Figures 1A,B).

#### Serum Immunoglobulin Levels

The serum IgG levels at time of diagnosis of CVID were significantly lower than those recorded for patients with HGG (3.10 ± 1.55 versus 6.32 ± 4.67 g/L, respectively, *p* < 0.0001; Figure 1D). However, serum IgG levels for CVID and HGG were both significantly lower than in patients with a diagnosis of IgGSCD and SpAD (*p* < 0.0001; Figure 1D). Serum IgA levels were also significantly reduced in CVID patients, compared with other PAD, consistent with the diagnostic criteria (0.23 ± 0.28 versus 1.16 ± 1.05 g/L, *p* = < 0.0001; Figure 1D). Serum IgM levels in CVID (0.3 ± 0.36 g/L) were significantly lower compared with HGG (0.97 ± 0.6 g/L; *p* < 0.0001), and IGSCD (1.15 ± 0.66 g/L; *p* < 0.0001) (Figure 1D). Serum Ig levels at time of diagnosis were not available for most of the XLA patients, due to the historic nature of their diagnoses.

#### Specific Antigen Responses

The results of pneumococcal polysaccharide vaccine responses were available for 55 patients with PAD reported here. For CVID, specific pneumococcal polysaccharide antibody titers were available for 30 of 116 patients. From these 30 records, 29 CVID patients were reported to have unprotective vaccine responses (96.7%). Reflecting the heterogeneity of these conditions, the single patient with protective vaccine responses, in retrospect had an atypical presentation for CVID with early-onset chronic mucocutaneous candidiasis, and subsequent adult-onset hypogammaglobulinemia, was identified to have a gain-of-function (GOF) *STAT1* mutation, which is not typically associated with PAD. Furthermore, consistent with the diagnostic criteria for CVID, a higher proportion of HGG and IGSCD patients demonstrated protective vaccine responses compared with the CVID and SpAD groups, who consistently demonstrated suboptimal vaccine responses (χ^2^; *p* < 0.001 and *p* < 0.01 respectively; Figure 1C).

#### Genetic Diagnoses

Although genetic testing was not uniformly used in the diagnosis of these patients, 40/178 had an identified genetic cause for PAD (Table S1 in Supplementary Material). This included 21 XLA patients with hemizygous *BTK* mutations. Of the remaining 19 patients with PAD and an identified genetic contribution, 17 had CVID, and 2 had IGSCD. Of the CVID patients, 4 had NFκB1 deficiency, and 3 were heterozygous for the (C104R) variant in *TNFSRF13B*, encoding TACI, a known risk-factor for the development of CVID (23). One of these patients also harbored a pathogenic mutation in *TCF3* (E555K), which causes an autosomal dominant form of agammaglobulinemia (24). In addition, we identified *NFKB2* mutations in three CVID and two IgGSCD patients, who had marked autoimmune clinical manifestations. Two patients initially diagnosed with CVID were found to have *CXCR4* mutations and three patients with CVID were rediagnosed with CTLA4 haploinsufficiency.

### Clinical Features and Disease Complications

**Figure 2:**
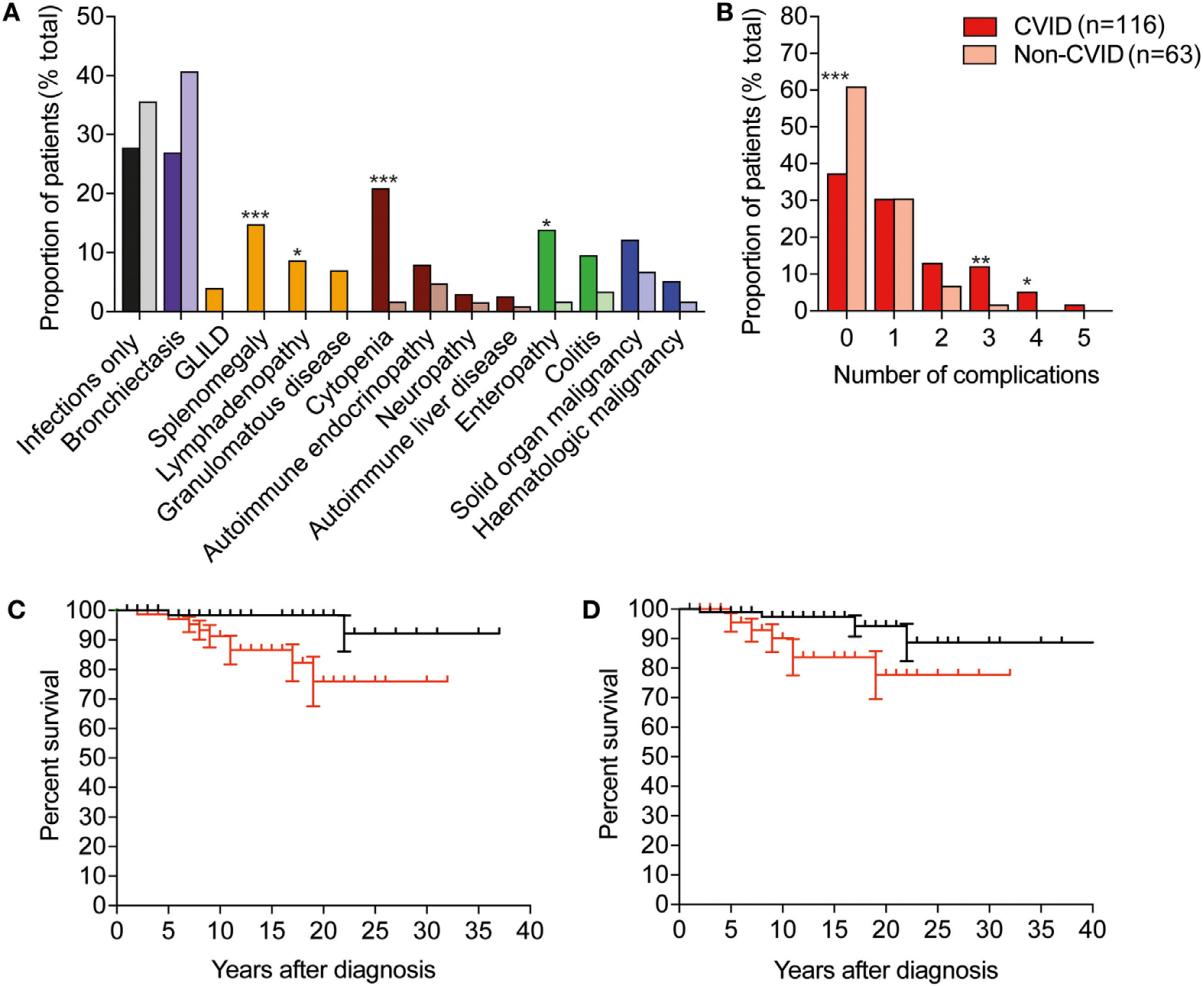
CVID is associated with more frequent non-infectious complications than other predominantly antibody deficiencies, and these complications are associated with reduced survival. **(A)** Histogram depicting frequency of complications in CVID, darker-colored columns, compared with non-CVID cases in lighter-colored columns. Abbreviations: GLILD, granulomatous lymphocytic interstitial lung disease; CVID, common variable immunodeficiency. Purple represents bronchiectasis; yellow represents non-malignant lymphoproliferative complications; brown, autoimmune complications; green, gastrointestinal disease, and blue, malignancies. (Fisher’s exact test: ***p < 0.001; **p < 0.01; and *p < 0.05). **(B)** Histogram depicting percentage of CVID and non-CVID patients with 0, 1, or more non-infectious complications of disease. **(C)** Survival after diagnosis in individuals with immune dysregulation is significantly reduced compared with patients with infections only (*p* < 0.05, Mantel–Cox log rank). Black line indicates survival of individuals without features of immune dysregulation, red line indicates survival of individuals with immune dysregulation. **(D)** Survival after diagnosis in individuals with bronchiectasis is significantly reduced compared with patients with infections only (*p* < 0.05, Mantel–Cox log rank). Black line indicates survival of individuals without bronchiectasis, and red indicates survival of individuals with bronchiectasis.

All patients presented with infections; however, most patients developed complications in addition; only 28% demonstrated an infection-only clinical phenotype (Table 2; Figure 2A). Respiratory tract involvement was the most common clinical manifestation followed by disease of the GI tract, skin and musculoskeletal system (Table 2; Figure 2A). Pneumonia and sinusitis were the most common infectious manifestations of the respiratory tract, present in 78% of all PAD patients, and GI infections were present in 19% of patients (data not shown). CVID patients were disproportionately affected and appeared susceptible to a broad range of infections that included *Giardia* in eight patients, *Campylobacter* and *Salmonella* in five, candidiasis in three, *Helicobacter pylori* in three and *Cryptosporidia* spp. in two patients. Bronchiectatic structural lung disease was most common in XLA with 66.7% patients affected compared with 27% of CVID patients (Table 2; Figure 2A). Four of these patients with chronic lung disease underwent lung transplantation; one female with CVID, and three males with XLA. One of four patients with Good syndrome also developed bronchiectasis.

**Table 2:** Complications in Victorian adults with predominantly antibody deficiency.

	CVID, *n*(%)	XLA	HGG	IGSCD	SpAD	Good syndrome	Total, *n*(%)
Total number of patients	116	21	22	12	4	4	179
Infections only^a^	33 (28)	7 (33)	12 (55)	4 (33)	1 (25)^c^	0	57 (32)
Bronchiectasis	31 (27)	14 (67)	4 (18)	4 (33)	2 (50)	1 (25)	28 (16)
GLILD	5 (4)	0	0	0	0	0	5 (3)
Autoimmunity (total)	44 (38)	0	3 (14)	4 (33)	1 (25)	2 (50)	54 (30)
Musculoskeletal	11 (9)	0	0	2 (17)	0	1 (25)	14 (8)
Cytopenia^b^	24 (21)	0	1 (5)	0	0	1 (25)^b^	26 (15)
Endocrine	9 (8)	0	0	2 (17)	1	0	12 (7)
Gastrointestinal disease (total)	27 (23)	0	2 (9)	1 (8)	0	0	30 (17)
Enteropathy	16 (14)	0	0	1 (8)	0	0	17 (9)
Colitis	11 (9)	0	2 (9)	0	0	1 (25)^c^	14 (8)
Granulomatous disease	8 (7)	0	0	0	0	1 (25)^c^	9 (5)
Autoimmune liver disease	3 (3)	0	1 (5)	0	0	0	4 (2)
Neuropathy	4 (3)	0	0	1 (8)	0	0	5 (3)
Malignancy^c^	20 (17)	0	2 (9)	3 (25)	0	2 (50)	27 (15)
Solid organ	14 (12)	0	2 (9)	2 (17)	0	1 (25)^c^	19 (11)
Hematological	6 (5)	0	0	1 (8)	0	1 (25)^c^	8 (4)

*CVID, common variable immunodeficiency; XLA, X-linked agammaglobulinemia; HGG, unclassified hypogammaglobulinemia; IGSCD, immunoglobulin subclass deficiency; GLILD, granulomatous lymphocytic interstitial lung disease; SpAD, specific antibody deficiency*.

*^a^Excludes autoimmunity, gastrointestinal disease, granulomatous disease, liver disease, malignancy, and GLILD*.

*^b^Excluding lymphopenia*.

*^c^Excluding thymoma*.

Neurologic manifestations were also evident in this cohort. There were five cases of meningitis, which were enterovirus-related in two patients, one of whom succumbed to a fatal progressive meningoencephalitis (Table 3). Neuropathy was reported in four CVID patients and a single patient with IGSCD (Table 2). The features of the neuropathy were not specified as to severity, pattern or progression. Seizures, optic neuritis, autoimmune hypophysitis were also recognized in individual patients from this cohort. Endocrine manifestations that were recorded included autoimmune thyroid disease in eight CVID patients, one IGSCD and one SpAD, while autoimmune adrenal insufficiency was reported in one CVID and one IGSCD patient.

**Table 3:** Causes of death among antibody deficient patients in Victoria (2000–2017).

Patient no.	Diagnosis	Sex	Clinical phenotype	Age at diagnosis	Age deceased	Cause of death
46	IGSCD^a^	F	Immune dysregulation	9	28	Enteroviral encephalitis
90	CVID	F	Infections only	62	70	Breast cancer
91	Good syndrome	M	Infections only	75	77	Thymoma
92	CVID	F	Immune dysregulation	69	74	Serous ovarian cancer
93	CVID	F	Infections only	47	74	Squamous esophageal cancer
113	CVID^b^	F	Immune dysregulation	67	81	Lung adenocarcinoma
123	CVID	F	Immune dysregulation	40	65	Intracerebral hemorrhage
156	CVID	M	Immune dysregulation	42	62	Diffuse large B cell lymphoma
157	CVID	F	Immune dysregulation	?	48	Sepsis
158	CVID	M	Immune dysregulation	71	78	Respiratory failure—ILD
159	XLA	M	Infections/bronchiectasis	?	58	Infection post lung transplant
160	CVID	F	Infections only	68	73	Not available
161	XLA	M	Infections/bronchiectasis	?	35	Lung transplant complication
162	CVID	M	Immune dysregulation	27	32	Enteropathy/cachexia/sepsis
163	XLA	M	Infections/bronchiectasis	13	59	Respiratory failure
164	XLA	M	Infections/bronchiectasis	?	39	Infection post lung transplant (CMV)
165	XLA	M	Infections/bronchiectasis/cirrhosis	?	36	HCV-related cirrhosis
166	CVID	M	Immune dysregulation	36	53	Respiratory failure and refractory enteropathy
167	CVID	F	Infections/bronchiectasis	71	80	Respiratory failure

*IGSCD, immunoglobulin subclass deficiency; CVID, common variable immunodeficiency; XLA, X-linked agammaglobulinemia; ILD, interstitial lung disease; CMV, cytomegalovirus; HCV, hepatitis C virus*.

*? indicates unknown*.

*^a^NFKB2 mutation*.

*^b^NFKB1 mutation*.

Overall, manifestations of immune dysregulation were most prevalent in CVID and IGSCD patients (72 and 67%, respectively), compared with only 5/22 hypogammaglobulinemic patients, and 1/4 SpAD (Table 2). The proportion of patients with all non-infectious manifestations was significantly higher in the CVID group, compared with the aggregated non-CVID cohort of patients (59/116 versus 12/38, respectively; χ^2^, *p* < 0.05).

We reviewed the incidence of specific clinical manifestations in the cohort according to diagnostic group (Figure 2A). Compared with the non-CVID antibody deficient patients, the 116 CVID patients more frequently developed cytopenia (*p* < 0.001), enteropathy (*p* < 0.01), splenomegaly (*p* < 0.01), lymphadenopathy (*p* < 0.05), and granulomatous infiltration (*p* < 0.05), but there were no significant differences between the proportions of patients with bronchiectasis, colitis, solid organ or hematological neoplasia and autoimmune liver disease.

To determine if organ complications were cumulative, we assessed the presence or absence of bronchiectasis, autoimmune liver disease, enteropathy, colitis, organomegaly, granulomas and malignancy across the key antibody deficient disease groups. Most patients suffered from <2 complications of disease; however, CVID patients were more likely to have >2 complications (Figure 2B).

### Management of PAD

Patients were identified on the basis of current or previous immunoglobulin replacement therapy. At the time of data analysis all patients with a diagnosis of XLA, CVID and Good syndrome were receiving replacement immunoglobulin and maintaining IgG mean trough levels of 8.8 g/L (data not shown). Antibiotics were used prophylactically in 10 of 20 CVID patients with available data while systemic immunosuppression was used in 17 CVID patients. These included nine individual patients treated with prednisolone, four patients with azathioprine or methotrexate in combination with prednisolone, whereas rituximab (anti-CD20) was administered in six CVID patients for cytopenias. One patient with CVID and granulomatous lymphocytic interstitial lung disease was treated with rituximab and mycophenolate. Splenectomy was performed on two patients for refractory cytopenia. One patient had undergone liver transplantation due to cirrhosis secondary to nodular regenerative hyperplasia and has tolerated tacrolimus well. One other patient had undergone renal transplantation. Three XLA patients and one CVID patient received lung transplants and were treated with combination immunosuppression including mycophenolate, tacrolimus and prednisolone as prophylaxis against rejection.

### Mortality

From this retrospective analysis, we identified 19 PAD patients who had died, including 4 from infections (cytomegalovirus, enteroviral encephalitis, sepsis-unspecified, and respiratory infection) and 6 patients died as a result of malignant disease (breast cancer, malignant thymoma, serous ovarian cancer, squamous esophageal cancer, lung adenocarcinoma, and diffuse large B cell lymphoma; Table 3). Of those, five had CVID and one had a diagnosis of Good syndrome, with a malignant thymoma. One patient died from an intracerebral hemorrhage, which was thought to be unrelated to PAD. Survival following diagnosis was significantly reduced in patients with immune dysregulation, with a 75% survival at 20 years after diagnosis compared with 98% for those with an infection-only phenotype (*p* < 0.05; Figure 2C). Significantly reduced survival was also noted in PAD patients with bronchiectasis as early as 10 years post-diagnosis (Figure 2D).

## Discussion

Primary immunodeficiencies are a heterogeneous mix of disorders, with >300 defined conditions. The most prevalent of these are PADs, which may present with a broad range of clinical features, age of onset, and population-dependent frequency. As a result, the underlying immunodeficiency diagnosis is typically significantly delayed or missed.

We aimed to characterize how PAD patients have been diagnosed, managed and manifested disease and sought to identify areas for improvement in clinical practice in Victoria, the second most populous state in Australia, with a population aged over 15 years of approximately 4.9 million at the end of 2016 (Australian Bureau of Statistics). Australia is a geographically expansive country, with a relatively small population; posing unique challenges for diagnosis and treatment of rare diseases. The most recent Australian study demonstrated incomplete capture of all PIDs, with the finding of 5.6 cases per 100,000 (5). We found a prevalence of PAD in Victorian adults of approximately 1 in 25,000, which is similar to other population frequencies internationally (11). The prevalence of PAD varies geographically, with a very high prevalence in Finland, a non-diverse population, in contrast to that of Australia, which is more heterogeneous (25).

As with any retrospective study, there are some limitations to this report. Data collection may be incomplete, and inconsistency may arise due to differences in practice between individual physicians and centers. Under-diagnosis is likely to relate to the relative infrequency of these conditions, and therefore lack of suspicion among primary care providers, particularly as the clinical features are varied (25). There is also a significant delay in diagnosis observed in the majority of these disorders, which is associated with an increased risk of mortality, and significant morbidity (26). Thus, increased awareness among medical practitioners and improvements in diagnostic tools are of great clinical importance. Implementation of increased awareness programs is likely to raise the number of patients to be diagnosed and potentially this will lead to identification of a higher prevalence of PAD than 1 in 25,000.

While this cohort represents a small group of patients, our median diagnostic delay of 9 years in the CVID group is longer than other recently reported cohorts. European data from more than 2,000 patients with CVID identified a median diagnostic delay overall of 5 years when the diagnosis was made prior to the year 2000, and 4.2 years when the diagnosis was made during or after the year 2000 (27). However, those patients comprised a large proportion of children, whereas our cohort only included adults, which may introduce a bias to a shorter diagnostic delay in that study. Indeed, in other smaller European adult cohorts a diagnostic delay of 7 years has been reported (28, 29).

Predominantly antibody deficiency remains a diagnostic challenge unless specifically considered in light of the varied non-infectious manifestations, and involvement with physicians with expertise in diagnosis and treatment of primary immunodeficiency. Our data demonstrate that distinguishing primary hypogammaglobulinemia from CVID cannot be readily made using laboratory parameters in isolation, as there is overlap in these parameters between the two conditions. Furthermore, vaccination studies, which are used for diagnosis of PAD, are complicated by the need for paired serum analyses, and are confounded by increasing community use of pneumococcal vaccines and variability in the measurement, and interpretation, of polysaccharide responses. Taken together, there is a need to improve PAD diagnosis, which may entail the use of molecular tests and dialog between internal medicine specialists and clinical immunologists.

Molecular tests that identify pathogenic mutations will improve diagnostic precision in PAD. In this cohort, patients who were later identified to have a genetic contribution to their disease, had a shorter time to a clinical diagnosis (Table 2). This may be influenced by the severity of presenting illnesses and/or a positive family history pre-empting screening for antibody deficiency. In addition, molecular tests may help identify patients-at-risk for certain disease complications, as in the case of *NFKB2* mutations which are associated with a high risk of central adrenal insufficiency. Further highlighting the complexities of clinical presentation in PID, we have described a patient who was initially misdiagnosed with CVID, but later found to have a *STAT1* GOF mutation after his diagnosis was reconsidered in light of early-onset mucocutaneous candidiasis.

We also identified one individual with digenic disease due to a pathogenic E555K variant in *TCF3* and C104R variant in *TNFRSF13B*. We have previously reported another kindred affected by PAD and autoimmune disease; the proband harbored a novel non-sense *TCF3* mutation and the C104R variant in *TNFRSF13B*, with a resultant CVID-like disorder and systemic lupus erythematosus (30). We are not aware of other reports of digenic PAD due to the more common pathogenic *TCF3* E555K variant in combination with an additional genetic risk-factor.

Our findings support improved access to diagnostic genomic studies in these patients, although currently the majority of CVID cases are unlikely to be readily diagnosed due to their complex pathogenesis (31, 32). Nevertheless diagnostic rates could increase to 30% for CVID patients selected for sequencing on the basis of certain clinical or laboratory features (14). Other studies have identified XLA in adult patients previously misdiagnosed as CVID, which has significant implications regarding missed opportunities for genetic counseling (33). The genetic diagnoses we have reported here may have been biased toward patients with more extreme phenotypes, and further work is ongoing to determine the clinical utility of genomic sequencing to aid diagnosis in the cohort more broadly.

Earlier diagnosis of PAD will facilitate earlier IgG replacement therapy and/or antibiotic therapy to mitigate the susceptibility to infections, and delay or prevent end-organ changes from infections such as bronchiectasis. However, optimal management of infections alone may not prevent complications that arise from immune dysregulation such as autoimmune disease, granulomatous inflammation and enteropathy, which affected 68% of our cohort. This is a higher proportion than reported in other cohorts and may reflect a referral bias toward patients with more severe disease, and a failure to include stable PID patients with infection-only phenotypes controlled on immunoglobulin replacement managed by non-Immunology services, or indeed those not receiving immunoglobulin at all.

Prediction of risk for the non-infectious complications of PAD is a major challenge. In a recent US study, unbiased network clustering using two large CVID patient datasets has identified novel biochemical markers of patients with lymphoproliferative, autoimmune and allergic disease (34). Whether these associations apply to the other PADs is unclear. In our cohort CVID patients suffered the most non-infectious complications, but we also identified non-infectious complications in other PADs. Non-infectious complications in IGSCD have also been recognized in other cohorts, although a recent Dutch study did not identify any non-infectious complications in a cohort of patients with HGG (35, 36). Importantly, two IGSCD patients in our cohort harbored *NFKB2* mutations, which may have predisposed these patients to the more severe clinical phenotype.

## Conclusion

Predominantly antibody deficiencies are the most common of the primary immune deficiency diseases, yet are still likely to be underdiagnosed, or diagnosed after the development of life-threatening complications. Individuals with PADs suffer from diverse and severe complications including infection, bronchiectasis and disorders of immune dysregulation, which are associated with reduced survival. These complications may be averted by earlier and more precise diagnosis, and targeted therapeutic interventions. Early diagnosis relies upon improved knowledge and appropriate clinical suspicion among internal medical physicians and primary care providers, as well as equitable access to genetic diagnosis and the benefits of personalized medicine.

## Ethics Statement

The study was carried out according to the principles of the Declaration of Helsinki and was approved by local human research ethics committees (Melbourne Health projects 2009.162, 2013.245; Walter and Eliza Hall Research Institute projects 10/02, 14/01; Alfred Health 109/15, 277/17). All living patients, or their next of kin, consented to the collection of their medical information. For those individuals who were deceased at the time of the data collection, ethical approval was obtained to review the medical records without the consent of the next of kin.

## Author Contributions

CS and JB designed the study, collected data, interpreted data, and wrote the manuscript. MZ and VB designed the study, interpreted the data, and wrote the manuscript; TG and EK collected and interpreted data. RS, PC, FH-L, MS, SH, SO, SB, GU, JL, MP, JM, KS, YT, PA, KN, RO, JD, and PH contributed data and assisted writing the manuscript.

## Conflict of Interest Statement

The authors declare that the research was conducted in the absence of any commercial or financial relationships that could be construed as a potential conflict of interest.
