# Supplementary material for: Delayed Diagnosis and Complications of Predominantly Antibody Deficiencies in a Cohort of Australian Adults
Source: Front Immunol. 2018 May 14;9:694. doi: 10.3389/fimmu.2018.00694 (PMC5960671; doi:10.3389/fimmu.2018.00694)
Supplement: Supplementary file 1 [file table_1.docx]

*Supplementary table: Symptom onset and diagnostic delay of patients with genetic causes of PADs*

| **Patient no.** | **Clinical diagnosis** | **Gene affected** | **Age at onset (years)** | **Age at diagnosis (years)** | **Diagnostic delay (years)** | **Total no. patients** | **Median diagnostic delay** |
| --- | --- | --- | --- | --- | --- | --- | --- |
| 30 | XLA | *BTK* | 0 | 0 | 0 | **21** | **2** |
| 38 | XLA | *BTK* | 0 | 0 | 0 |  |  |
| 39 | XLA | *BTK* | 0 | 0 | 0 |  |  |
| 40 | XLA | *BTK* | 0 | 0 | 0 |  |  |
| 41 | XLA | *BTK* | 1 | 0 | 0 |  |  |
| 116 | XLA | *BTK* | 0 | 1 | 1 |  |  |
| 120 | XLA | *BTK* | 0 | 4 | 4 |  |  |
| 122 | XLA | *BTK* | 0 | 0 | 0 |  |  |
| 122 | XLA | *BTK* | 0 | 0 | 0 |  |  |
| 125 | XLA | *BTK* | 0 | 0 | 0 |  |  |
| 126 | XLA | *BTK* | 0 | 3 | 3 |  |  |
| 132 | XLA | *BTK* | 0 | 0 | 0 |  |  |
| 133 | XLA | *BTK* | 0 | 3 | 3 |  |  |
| 159 | XLA | *BTK* | ? | ? | ? |  |  |
| 161 | XLA | *BTK* | 0 | 15 | 15 |  |  |
| 163 | XLA | *BTK* | 0 | 0 | 0 |  |  |
| 164 | XLA | *BTK* | 0 | 0 | 0 |  |  |
| 165 | XLA | *BTK* | ? | ? | ? |  |  |
| 164 | XLA | *BTK* | ? | ? | ? |  |  |
| 165 | XLA | *BTK* | 1 | 3 | 2 |  |  |
| 169 | XLA | *BTK* | ? | ? | ? |  |  |
| 46 | IGSCD | *NFKB2* | 2 | 9 | 7 |  | |
| 47 | IGSCD | *NFKB2* | 2 | 7 | 5 |  |  |
| 48 | CVID | *NFKB2* | 27 | 38 | 11 |  |  |
| 49 | CVID | *NFKB2* | 1 | 5 | 4 |  |  |
| 59 | CVID | *NFKB2* | 13 | 17 | 4 | **5** | **8** |
| 45 | CVID | *NFKB1* | 14 | 27 | 13 | **4** | **7** |
| 112 | CVID | *NFKB1* | 31 | 39 | 8 |  |  |
| 113 | CVID | *NFKB1* | 54 | 67 | 13 |  |  |
| 114 | CVID | *NFKB1* | 27 | 28 | 1 |  |  |
| 84 | CVID^#^ | *CXCR4* | 2 | 27 | 25 | **2** | **13** |
| 85 | CVID^#^ | *CXCR4* | 3 | 4 | 1 |  |  |
| 103 | CVID | *TCF3** | 1 | 2 | 1 | **1** | **N/A** |
| 56 | CVID | *TACI*^‡^ | 36 | 41 | 5 | **3** | **2.5** |
| 83 | CVID | *TACI*^‡^ | 44 | 46 | 2 |  |  |
| 119 | CVID | *CTLA4* | 6 | 16 | 10 | **3** | **7** |
| 141 | CVID | *CTLA4* | 15 | 19 | 4 |  |  |
| 120 | CVID | *CTLA4* | 7 | 13 | 6 |  |  |
| 19 | CVID | *STAT1* | 16 | 35 | 19 | **1** | **N/A** |
| 125 | CVID | *PI3KCD* | 28 | 47 | 16 | **1** | **N/A** |

*(CVID, Common Variable Immunodeficiency; XLA, X-linked agammaglobulinemia; ?, indicates unknown; IGSCD, Immunoglobulin subclass deficiency;*

*#, diagnosis changed to WHIM syndrome after genetic testing; *, patient also harbors TACI variant;* ^‡^*, C104R variant; TACI, encoded by gene TNFRSF13B)*
